# Reduction of Bladder Cancer Chemosensitivity Induced by the Effect of HOXA-AS3 as a ceRNA for miR-455-5p That Upregulates Notch1

**DOI:** 10.3389/fonc.2020.572672

**Published:** 2021-02-12

**Authors:** Dajin Chen, Shangzhi Xie, Ying Wu, Yu Cui, Ying Cai, Lan Lan, Hao Yang, Jianghua Chen, Wei Chen

**Affiliations:** ^1^Kidney Disease Center, the First Affiliated Hospital, School of Medicine, Zhejiang University, Hangzhou, China; ^2^Department of Medical Oncology, Tongde Hospital of Zhejiang Province, Hangzhou, China; ^3^Cancer Institute of Integrated Traditional Chinese and Western Medicine, Key Laboratory of Cancer Prevention and Therapy Combining Traditional Chinese and Western Medicine of Zhejiang Province, Zhejiang Academy of Traditional Chinese Medicine, Tongde Hospital of Zhejiang Province, Hangzhou, China

**Keywords:** bladder cancer, HOXA-AS3, EMT, drug sensitivity, Notch1

## Abstract

Chemoresistance is one of the main causes of recurrence in bladder cancer patients and leads to poor prognosis. Recently, long non-coding RNAs, like HOXA-AS3, have been reported to regulate chemoresistance in several types of cancer. In this study, we aimed to determine whether HOXA-AS3 can mediate cisplatin resistance in bladder cancer, and its potential mechanism of action. We determined the viability, proliferation, and apoptosis of bladder cancer cells using a CCK-8 assay, EdU staining, and flow cytometry, respectively. We used western blot analysis to assess the expression of markers of epithelial-mesenchymal transition (EMT) and Notch1. We then confirmed expression of these EMT-related markers by immunofluorescence analysis. We found that hypoxia promoted resistance to cisplatin and upregulated the level of HOXA-AS3 in BC cells. Inhibition of HOXA-AS3 enhanced hypoxia-induced cisplatin sensitivity by regulating EMT and Notch1 in BC cells. A dual-luciferase reporter assay confirmed that HOXA-AS3 directly targets miR-455-5p and that Notch1 was a potential target of miRNA-455-5p. We also found that the positive effect of HOXA-AS3 inhibition on cisplatin resistance and tumorigenesis was alleviated when BC cells were transfected with miR-455-5p. Finally, we showed combining HOXA-AS3 small interfering RNA (siRNA) with cisplatin treatment inhibited tumorigenesis in a BALB/c nu/nu mouse model. Our findings indicate that HOXA-AS3 may function as a competing endogenous RNA (ceRNA) of miR-455-5p to regulate Notch1 and play an important role in regulating chemotherapeutic drug sensitivity in BC cells. Therefore, HOXA-AS3 may be a novel therapeutic target for treating bladder cancer.

## Introduction

Bladder cancer (BC) is ranked as the ninth most common cancer worldwide, and men are more than three times more likely to develop the disease than women (1, 2). Cisplatin is extensively used in the clinic as a first-line treatment for many cancers, including liver, ovarian, non-small cell lung cancer (NSCLC), and BC (3–5); however, its efficacy is limited due to severe side effects and the development of drug resistance (6). Indeed, most patients become irresponsive to cisplatin and eventually die of the disease (7). Therefore, understanding the mechanisms behind cisplatin resistance is key for improving BC treatment and prognosis.

Long non-coding RNAs (lncRNA), a group of evolutionarily conserved RNAs longer than 200 nucleotides, modulate gene expression at the epigenetic, transcriptional, and post-transcriptional levels (8). Recent studies have shown abnormal expression of lncRNA in many types of tumors, playing important roles in cancer progression, including cell proliferation, differentiation, invasion, and metastasis (9, 10). Importantly, lncRNAs are considered key regulators in drug resistance, and may also act as promising prognostic and therapeutic targets. Several studies have shown lncRNAs may interact with miRNAs and mutually modulate their expression (11, 12). They may function as competing endogenous RNAs (ceRNAs) with miRNAs and subsequently regulate gene expression by post-transcriptional silencing of the target RNAs (13, 14). Indeed, the interaction of lncRNAs and miRNAs may provide new insight into cancer biology. For instance, knockdown of the lncRNA SBF2-AS1 increased the chemosensitivity of gemcitabine through inhibiting expression of the twinfilin actin binding protein 1 (TWF1) by competitively binding to miR-142-3p in pancreatic cancer (15). In addition, the absence of the lncRNA FOXD2-AS1 enhanced cisplatin sensitivity of NSCLC cells by regulating the miR-185-5p-SIX1 axis (16). Therefore, understanding the functions of lncRNAs in cancer has become an area of extensive research.

HOXA-AS3 belongs to the clusters of HOX genes, a group of highly homologous transcription factors that regulate embryological development, and also regulate hematopoietic lineage and differentiation (17, 18). There is mounting evidence that lncRNAs, such as HOX-AS3, regulate cancer cell growth, metastasis, and resistance to chemotherapy in several types of tumors (19), and this makes them promising targets for novel cancer therapies (20). Previous studies have shown that overexpression of the lncRNA HOXA‐AS3 promotes tumor progression and is a predictor of poor prognosis in glioma (21). Increased HOXA‐AS3 levels were also found to promote proliferation in lung adenocarcinoma (22). However, the mechanisms of these lncRNAs in the various types of cancer can vary. For instance, knockdown of the lncRNA TUG1 promoted cisplatin sensitivity in an esophageal squamous cell carcinoma cell line (TE-1) by regulating nuclear factor-like 2 (Nrf-2) expression (23). Further, inhibition of the lncRNA HOTAIR enhanced doxorubicin sensitivity in breast cancer by downregulating the PI3K/AKT/mTOR pathway (24). Based on these findings, we sought to unravel the role of HOXA-AS3 in BC.

Hypoxia plays a role in resistance to chemotherapeutic treatments in several cancers (25–27). It is commonly present in the microenvironment of solid tumors and is associated with tumor invasion, distant metastasis, and epithelial–mesenchymal transition (EMT) (28–30). HOXA-AS3 inhibition has been shown to induce epithelial-mesenchymal transition (EMT) in NSCLC, and increase its resistance to cisplatin (31). EMT is a critical mechanism of cancer metastasis. During the EMT process, cancer cells lose their epithelial features and acquire a mesenchymal phenotype, and thus obtain enhanced metastatic ability (32). It has been reported that inhibition of SNHG7 could promote tumor growth and the EMT phenotype *via* upregulation of the targets of miR-34a, including Notch1 (33).Therefore, lncRNAs such as HOXA-AS3 may modulate EMT in various types of cancer, including BC, by regulating the expression of Notch1. Indeed, HOXA-AS3 knockdown was previously shown to inhibit the proliferation, metastasis, and EMT in hepatocellular carcinoma (HCC) cells *via* the MEK/ERK signaling pathway (34). Thus, HOXA-AS3 may regulate EMT in BC in a similar manner, i.e., by interacting with miRNAs or altering the expression of proteins such as Notch1. However, the effect of HOXA‐AS3 on drug sensitivity (e.g., to cisplatin) and the regulation of EMT in BC are yet to be explored.

In this study, we examined whether HOXA-AS3 mediates cisplatin resistance in BC cells, and then unraveled its underlying mechanism(s) of action.

## Materials and Methods

### Cell Culture and Human Tissues

Human bladder cancer cells (UM-UC-3, J82, and BIU-87) were purchased from ATCC. All cells were cultured in RPMI 1640 medium (Gibco, Grand Island, NY, USA) supplemented with 10% fetal bovine serum (FBS) and 1% penicillin/streptomycin, maintained in humidified air containing 5% CO_2_ at 37°C. The tumor tissue samples of BC patients were obtained from First Affiliated Hospital of Zhejiang University. All patients received written informed consent and the study protocol was approved by the Clinical Research Ethics Committee of the First Affiliated Hospital of Zhejiang University.

### Cell Viability

A cell counting kit-8 (CCK-8; Dojindo, Kumamoto, Japan) assay was used to determine cell viability. Briefly, BC cells were plated into 96-well plates at a density of 3 × 10^3^ cells/well and incubated overnight at 37°C. Subsequently, cells were treated with a series of concentrations of cisplatin (0, 0.625, 1.25, 2.5, 5, or 10 μM) for 48 h or transfected with HOAXA-AS3 siRNA, miR-455-5p inhibitor, or HOXA-AS3 siRNA plus miR-455-5p inhibitor for 48 h. Then, 10 μl of CCK-8 solution was added to each well and cultured for 3 h at 37°C before the absorbance at 450 nm was measured using an MRX II microplate reader (Dynex Technologies, Chantilly, USA). Relative cell viability was calculated as a percentage of the untreated controls.

### Cell Transfection

LncRNA HOXA‐AS3 siRNA, plasmid, and negative control siRNA were constructed by GenePharma (Shanghai, China). MiR‐455-5p mimic (80 nM), inhibitor (80 nM), negative control (NC) mimic, and NC inhibitor were prepared by Ribo Biotechnology (Guangzhou, China). Transfections of these siRNAs, mimics, inhibitors, or plasmid were conducted using Lipofectamine 2000 (Invitrogen, Carlsbad, CA) following the manufacturer’s instructions. The HOXA-AS3 plasmid was shown in **Table 1**.

**Table 1:** Plasmid profile of B3442 PEX-3-HOXA-AS3.

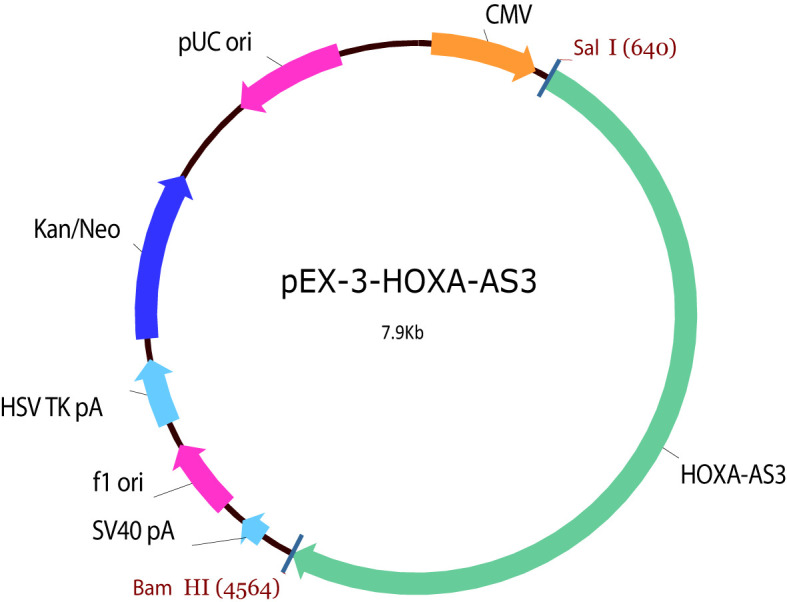	

HOXA-AS3 siRNA:

5′-UCUAUUCUCGCAAGGGAAATT-3′

5′-UUUCCCUUGCGAGAAUAGATT-3′

miR-455-5p mimics:

5’–GCAGTCCATGGGCATATACAC–3’;

miR-455-5p inhibitor:

5’–GTGTATATGCCCATGGACTGC–3’.

### Western Blot Analysis

Briefly, 40 μg proteins/well were resolved by 10% sodium dodecyl sulfate polyacrylamide gel electrophoresis (SDS-PAGE) and transferred to PVDF (Millipore, Darmstadt, Germany) membranes. After 1 h of blocking, membranes were incubated with the following primary antibodies overnight at 4°C: Notch1, E-cadherin, and Vimentin (Abcam; 1:1,000 dilution). After washing, the membranes were incubated with secondary antibodies (Cell Signaling Technology, USA). Finally, the bands were detected using electrochemiluminescence (ECL; Pierce, Rockford, Illinois, USA) and visualized using Image Lab 5.0 (Bio-Rad, Hercules, CA, USA).

### Quantitative Real-Time PCR Analysis

Total RNA from BC cells was extracted by TRIzol reagent (Invitrogen, Carlsbad, CA) and RNA from paraffin specimens was extracted by RNeasy FFPE Reagent kit (Qiagen, Hilden, Germany) according to the manufacturer’s protocol . Then, messenger RNA (mRNA) was reverse transcribed using SuperScript TM II Reverse Transcriptase (Invitrogen, Carlsbad, CA), and cDNA detected using SYBR@ Premix Ex TaqTM (TaKaRa Bio Group, Shiga, Japan). To quantify miRNAs, we first used a miScript reverse transcription kit (Qiagen, Hilden, Germany), followed by amplification using SYBR@ Premix Ex TaqTM (Takara). B-actin and U6 RNA were used as internal loading controls for mRNAs and miRNAs, respectively. The primers used for qRT-PCR were as follows: HOXA-AS3 forward: 5’-CACCTCTCTCATCGAAAAACCG-3’; and reverse: 5’-GCACCAGGAAAGAGGACAATTC-3’; miR-455-5p:5’-TATGTGCCTTTGGACTACATCG-3’.

### Luciferase Assay

HOXA-AS3-3’UTR wild type or mutant reporter plasmids (which included the binding sequence for miR-455-5p or the mutated binding sites, respectively) were constructed by GenePharma. The plasmids were then transiently co-transfected into cells with luciferase reporter vectors with the miR-455-5p-mimic, miR-455-5p inhibitor, or control using Lipofectamine 2000. After transfection for 48 h, the relative luciferase activity of the wild type or mutant HOXA-AS3-3’-UTR was measured with a dual-luciferase reporter assay (Promega, USA).

### Flow Cytometry Analysis

The number of apoptotic cells was determined by an Annexin V-FITC Apoptosis Detection Kit (Abcam). In brief, cells (treated as above) were harvested by trypsinization, rinsed with ice-cold phosphate PBS, and centrifuged to remove the supernatant. Then, the cells were resuspended in 100 μl 1× binding buffer and stained with 5 μl annexin V and 5 μl propidium iodide (PI) for 15 min at room temperature in the dark. Finally, the percentage of apoptotic cells was determined using a flow cytometer (LSRII, BD Biosciences, Franklin Lakes, NJ, USA)

### Cell Proliferation Assay

Cell proliferation was determined using a Click-iT^®^ EdU Imaging Kit according to the manufacturer’s (Invitrogen) protocols.

### Immunofluorescence Analysis

NSCLC cells were seeded into 48-well plates at a density of 3 × 10^3^ cells/well. Cells were fixed with 4% formaldehyde for 15 min, washed with PBS, treated with 5% bovine serum albumin (BSA) for 30 min at room temperature, and incubated with anti-E-cadherin (1:200) or anti-human vimentin (1:200) primary antibodies (Cell Signaling Technology, Danvers, MA, USA) at 4°C overnight. The cells were incubated with a FITC-conjugated anti-rabbit secondary antibody (Abcam, Cambridge, USA) at 4°C for 2 h. Nuclear staining was performed with DAPI (Sigma, St. Louis, MO, USA) at room temperature for 5 min. Following two washes with PBS, cells were observed using an inverted fluorescence microscope (Olympus, Tokyo, Japan).

### Nude Mouse Xenograft Model

Female BALB/c nu/nu mice (4–5 weeks old) were purchased from Shanghai SLAC Laboratory Animal Co., Ltd (Shanghai, China). UMUC-3 cells were subcutaneously injected into the left hip of three mice. After the tumor was formed, a small section (1 mm^3^) of tumor tissue was inoculated into the experimental group nude mice. After 10 days, the tumors had a diameter of 0.5 cm and reached a volume of ~50–100 mm^3^. The mice were randomly divided into four groups (n = 16 per group) : control, HOXA-AS siRNA (2 nmol), cisplatin (2.5 mg/kg), or HOXA-AS3 siRNA plus cisplatin. HOXA-AS3 siRNA was injected intratumorally four times from day 0 to 14, while cisplatin was injected into the tail vein once every 2 days for 2 weeks. Tumor volumes were recorded every 2 days and body weight was measured daily. The tumor volume (mm^3^) was calculated using the formula V = (length × width^2^/2). 8 8 mice per group were sacrificed humanely on day 15 after treatment, the resected tumors were weighed and observe the survival of the remaining mice. All experimental protocols were approved by the Medical Ethics Committee of the First Affiliated Hospital of Zhejiang University. The experimental procedures conformed to the National Institutes of Health Guide for Care and Use of Laboratory Animals (NIH Publications, No. 8023, revised 1978).

### Immunocytochemistry

Immunohistochemical staining was performed on paraffin-embedded mouse tissue sections (5 mm) to determine Ki-67 and Notch1 expression. The slides were incubated with anti–Ki-67 and anti-Notch1 antibodies (1:500, Abcam) overnight at 4°C. A horseradish peroxidase (HRP) detection system (ZSGB-Bio, Beijing, China) and the diaminobenzidine (DAB) substrate kit (ZSGB-Bio) were used as detection reagents. After counterstaining with hematoxylin (ZSGB-Bio), the sections were dehydrated and mounted, and observed under a light microscope (Olympus, Tokyo, Japan). The positive rates were measured using Image-Pro Plus v.6.0 software (Media Cybernetics, Bethesda, MD, USA).

### Terminal Deoxynucleotidyl Transferase dUTP Nick End Labeling

TUNEL was used to identify apoptosis in paraffin-embedded mouse tissue sections (5 mm) with an *in situ* cell death detection kit (Roche, Basel, Switzerland) according to the manufacturer’s instructions. The apoptotic cells were observed under a light microscope (Olympus, Tokyo, Japan). The assay was independently repeated three times. The positive rates were measured using Image-Pro Plus v.6.0 software.

### Statistical Analysis

The experimental data are presented as means ± standard deviation (SD). Statistical analysis was performed using GraphPad Prism 7 (GraphPad, San Diego, CA, USA). The significance of differences between two groups was analyzed by Student’s t-test and multiple group comparisons were analyzed with one-way ANOVA. *P < 0.05, **P < 0.01, and ***P < 0.001 were considered significant.

## Results

### Hypoxia Induced a Reduction in Chemosensitivity to Cisplatin and Upregulation of HOXA-AS3 in BC Cells

**Figure 1:**
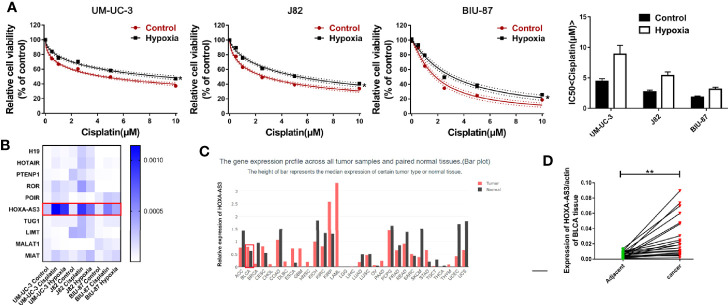
Hypoxia induces cisplatin resistance and upregulation of HOXA-AS3 in bladder cancer (BC) cells. **(A)** CCK-8 was used to determine cell viability after treatment with cisplatin under normoxic and hypoxic conditions. *P < 0.05 *vs.* control. **(B)** The level of long non-coding RNAs (lncRNAs) after cisplatin treatment under hypoxic or normoxic conditions was examined by qRT-PCR. **(C)** Gene expression profiling interactive analysis (GEPIA) database analysis of HOXA-AS3 expression in bladder cancer tumor samples and paired normal tissues. **(D)** Quantitative RT-PCR analysis of HOXA-AS3 levels in bladder cancer tissue and adjacent normal tissue.

Hypoxia has been found to induce chemotherapy resistance in various tumors and cancer cells (25, 26). Accordingly, the present findings from CCK-8 analysis indicated that hypoxia could induce resistance of BC cells to cisplatin, as indicated by the IC50 values in the histogram in **Figure 1A**. Next, we used qRT-PCR to detect the level of a series of lncRNAs after cisplatin treatment under hypoxic or normoxic conditions. We found that HOXA-AS3 expression was significantly increased under hypoxic conditions in BC cells **(****Figure 1B**). Using gene expression profiling interactive analysis (GEPIA), we analyzed HOXA-AS3 expression across all tumor samples and paired normal tissues and found that HOXA-AS3 was significantly upregulated in bladder tumor tissues compared with normal tissues (**Figure 1C**). We also analyzed the clinical analysis of the relationship between HOXA-AS3 expression and the clinicopathological parameters in bladder cancer, showing that high HOXA-AS3 expression in 30 bladder cancer patients was closely related to large tumor size (P=0.0123), the advanced TNM stage (P=0.0173), and invasion(muscle) (P=0.0352) (**Table 2****)**. Furthermore, qRT-PCR confirmed that the level of HOXA-AS3 in cancer tissue was higher than that in adjacent tissue (**Figure 1D**). We also confirmed that HOXA-AS3 expression was gradually upregulated with increasing cisplatin concentrations using qRT-PCR analysis (**Supplemental Figures 1A**). Therefore, hypoxia upregulated HOXA-AS3 and appears to reduce chemosensitivity to cisplatin in BC cells.

**Table 2:** The correlations between HOXA-AS3 and clinical characteristics of bladder cancer patients.

Clinical parameters	N = 30		HOXA-AS3 expression		p-value
			High	Low	
Sex	Female	9	3	6	0.6256
	Male	21	9	12	
Age(year)	<70	17	6	9	0.3096
	≥70	13	7	6	
Stage	I–II	19	7	12	0.0173*
	III–IV	11	9	2	
Tumor size (cm)	<3	17	4	13	0.0123*
	≥3	13	9	4	
Invasion (muscle)	Yes	12	8	4	0.0352*
	NO	18	5	13	
Lymphatic metastasis	Yes	8	4	v	0.6568
	NO	22	13	9	
Distant metastasis	Yes	5	4	v	0.1017
	NO	25	10	15	
Histological grade	High	13	7	6	0.7851
	Low	17	10	7	

### Downregulation of HOXA-AS3 Enhances Cisplatin Sensitivity Under Normoxic and Hypoxic Conditions

**Figure 2:**
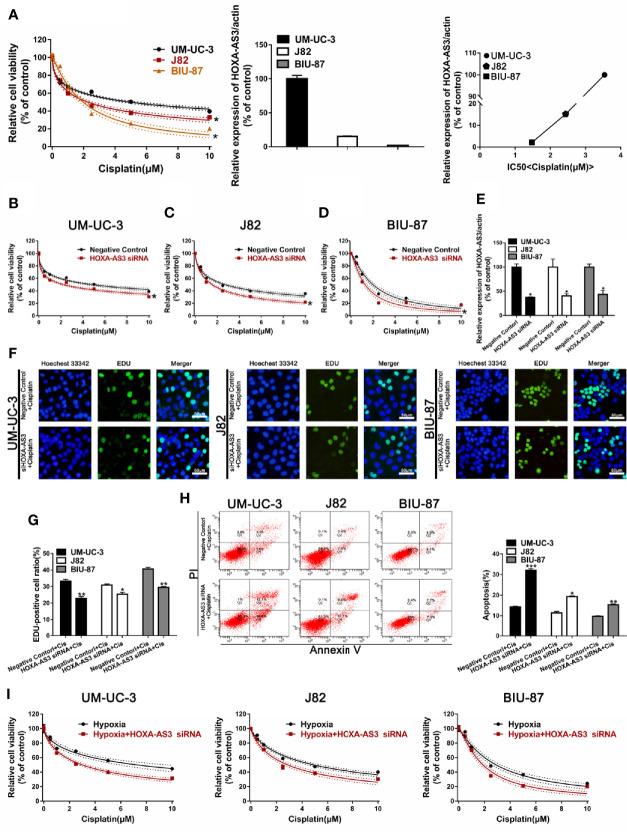
Downregulation of HOXA-AS3 enhances cisplatin sensitivity. **(A)** Quantitative RT-PCR analysis of HOXA-AS3 levels in three bladder cancer cell lines. Cell viability (CCK-8) analysis of bladder cancer cells treated with various concentrations of cisplatin *P < 0.05 *vs.* UM-UC-3. Correlation curve showing the association of the expression of long non-coding RNA (lncRNA) HOXA-AS3 with sensitivity to cisplatin in bladder cancer (BC) cells. **(B**–**D)** Cell viability (CCK-8) analysis of bladder cancer cells transfected with HOXA-AS3 small interfering RNA (siRNA) or a negative control followed by treatment with different concentrations of cisplatin (0, 0.625, 1.25, 2.5, 5, 10 μM). *P < 0.05 *vs.* negative control. **(E)** The interference efficiency of HOXA-AS3 siRNA was determined by qRT-PCR. *P < 0.05, **P < 0.01. **(F**, **G)** Photomicrographs and bar graphs depicting EdU staining and relative EdU-positive ratio in bladder cancer cells after treatment with cisplatin alone, or cisplatin combined with HOXA-AS3 siRNA. *P < 0.05, **P < 0.01. **(H)** The number of apoptotic cells in bladder cancer cells transfected with a negative control or HOXA-AS3 siRNA followed by cisplatin treatment, as detected by flow cytometry. *P < 0.05, **P < 0.01, ***P < 0.001 *vs.* negative control plus cisplatin. **(I)** Knockdown of HOXA-AS3 siRNA enhanced cisplatin sensitivity under hypoxic conditions.

We showed BIU-87 had the lowest HOXA-AS3 expression among all three human bladder cancer cell lines tested **(****Figure 2A****)**. We also showed BIU-87 was more sensitive to cisplatin than J82 and UM-UC-3 cells, indicating the level of HOSA-AS3 was negatively correlated to cisplatin sensitivity in BC cells **(****Figure 2A****)**. Next, we examined the effect of HOXA-AS3 on cisplatin sensitivity and its potential mechanism of action in BC cells. Using a CCK-8 cell viability assay, we found all three BC cell lines that were transfected with HOXA-AS3 siRNA had increased cisplatin sensitivity compared with the negative controls (**Figures 2B–E**), while the HOXA-AS3 plasmid had reduced cisplatin sensitivity (**Supplemental Figures 2A–D**). These results were confirmed by EdU staining analysis of cell proliferation (**Figures 2F, G**). qRT-PCR confirmed the expression of HOXA-AS3 following transfection with or without HOXA-AS3 siRNA (**Figure 2E**). Finally, as cisplatin-induced refractoriness to apoptosis is an important characteristic of chemoresistance, we examined the effect of HOXA-AS3 on apoptosis using flow cytometry. As shown in **Figure 2H**, combining HOXA-AS3 siRNA and cisplatin treatment significantly increased the rate of apoptosis of BC cells. Furthermore, knockdown of HOSX-AS3 could reverse hypoxia-induced cisplatin resistance **(****Figure 2I****)**. Together, these findings indicate that HOX-AS3 may regulate cisplatin sensitivity in BC cells under normoxic and hypoxic conditions.

### HOXA-AS3 Regulates Notch1 Expression and Reverses Hypoxia-Induced EMT in BC Cells

**Figure 3:**
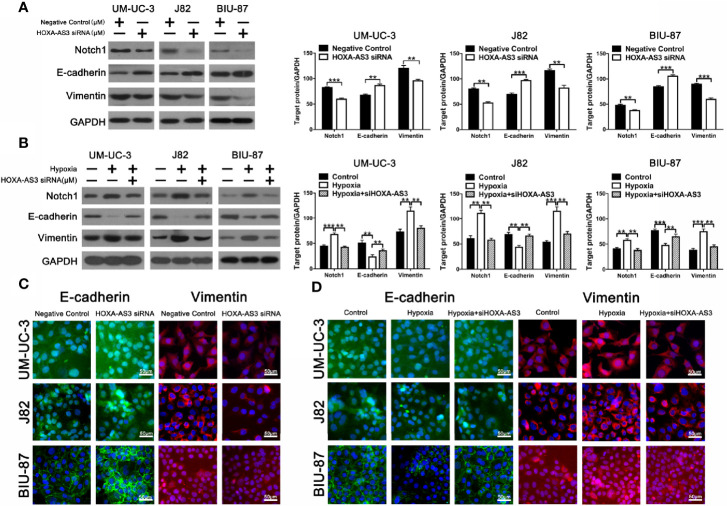
HOXA-AS3 regulates Notch1 expression and reverses hypoxia-induced EMT in bladder cancer (BC) cells. **(A**, **B)** Protein levels of EMT markers and Notch1 in bladder cancer cells transfected with a negative control or HOXA-AS3 small interfering RNA (siRNA) under normoxic or hypoxic conditions, by western blot analysis. **P < 0.01, ***P < 0.001. **(C**, **D)** Immunofluorescence analysis of E-cadherin and vimentin expression in BC cells transfected with a negative control or HOXA-AS3 siRNA under normoxic or hypoxic conditions.

Increasing evidence shows EMT plays an important role in regulating proliferation, migration, and chemoresistance in cancer cells (35, 36). Recent studies have reported that HOX-AS3 is closely related to cisplatin resistance and EMT. Therefore, we examined levels of EMT-related proteins in BC cells transfected with either HOXA-AS3 or HOXA-AS3 siRNA. Using western blot, we found that HOXA-AS3 siRNA treatment upregulated the expression of E-cadherin and downregulated Notch1 and vimentin expression in BC cells. Furthermore, transfection with HOXA-AS3 siRNA reversed the effects of hypoxia by upregulating E-cadherin expression and downregulating Notch1 and vimentin expression **(****Figures 3A, B****)**. Immunofluorescence analysis of E-cadherin and vimentin expression was consistent with these results **(****Figures 3C, D****)**. These findings indicate that inhibition of HOXA-AS3 could reverse hypoxia-induced EMT in BC cells.

### HOXA-AS3 Targets miR-455-5p

**Figure 4:**
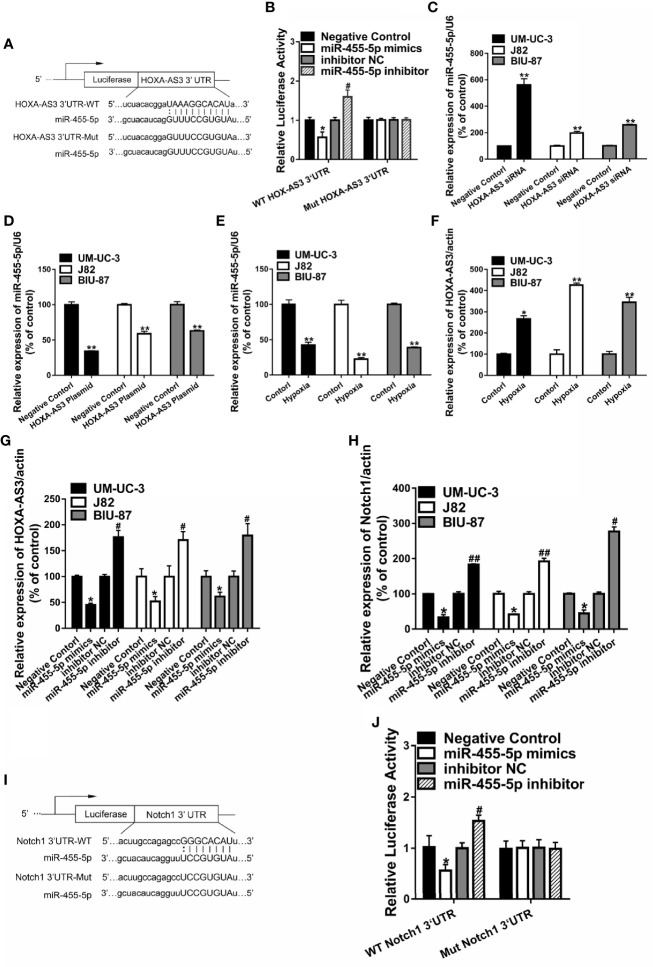
HOXA-AS3 may function as a competing endogenous RNA of miR-455-5p. **(A)** The predicted binding site between HOXA-AS3 and miR-455-5p and the mutation in the predicted seed region. **(B)** Relative luciferase activity of bladder cancer (BC) cells co-transfected with WT-HOXA-AS3 or Mut-HOXA-AS3 and NC, inhibitor NC or miR-455-5p mimics, and inhibitor. *P < 0.05 *vs.* NC; ^#^P < 0.05 *vs.* inhibitor NC. **(C**, **D)** Expression of miR-455-5p in BC cells transfected with HOXA-AS3 small interfering RNA (siRNA) or HOXA-AS3 detected by qRT-PCR. **P < 0.01. **(E**, **F)** qRT-PCR results showing the level of miR-455-5p and HOXA-AS3 under hypoxic or control conditions. *P < 0.05, **P < 0.01. **(G)** Expression of HOXA-AS3 in BC cells transfected with the miR-455-5p mimic or inhibitor detected by qRT-PCR. *P < 0.05 *vs.* negative control (NC); ^#^P < 0.05 *vs.* inhibitor NC. **(H)** Expression of Notch1 in BC cells transfected with the miR-455-5p mimic or inhibitor, as detected by qRT-PCR. *P < 0.05 *vs.* negative control (NC); ^#^P < 0.05, ^##^P < 0.01 *vs.* inhibitor NC. **(I**, **J)** Relative luciferase activity of BC cells co-transfected with WT-Notch1 or Mut-Notch1 and NC, inhibitor NC or miR-455-5p mimics, and inhibitor. *P < 0.05 *vs.* negative control (NC); ^#^P < 0.05 *vs.* inhibitor NC.

Recent studies suggest that lncRNAs could act as a sponge or decoy and compete with other genes for miRNA binding, and thereby, reduce the regulatory effect of miRNAs on their target mRNAs (11). In order to determine whether HOXA-AS3 brings about its effects by interacting with miRNA, we examined the effect of cisplatin on all the miRNAs deposited in the RegRNA2.0 database (http://regrna2.mbc.nctu.edu.tw/). We examined the level of all the miRNAs after treatment with or without cisplatin in BC cells and found that miR-455-5p was significantly downregulated after cisplatin treatment (**Supplemental Figure 3A**). This indicates that HOXA-AS3 may interact with miR-455-5p *via* complementary base pairing. Therefore, we constructed two versions of HOXA-AS3, that is, WT-HOXA‐AS3 and Mut-HOXA‐AS3, and used a luciferase reporter assay to verify the interaction between HOXA‐AS3 and miR‐455-5p. The 3′UTR residues predicted to interact with miR-455-5-p were mutated in Mut-HOXA‐AS3 (**Figure 4A**). We found that the addition of an miR‐455-5p mimic considerably reduced the luciferase activity of WT-HOXA‐AS3, while the addition of an miR‐455-5p inhibitor increased its activity (**Figure 4B**). However, neither the miR‐455-5p mimic nor the miR‐455-5p inhibitor had any impact on the luciferase activity of Mut-HOXA‐AS3 (**Figure 4B****)**. Furthermore, miR-455-5p expression was significantly upregulated following transfection with HOXA-AS3 siRNA, while it was downregulated after transfection with the HOA-AS3 plasmid in BC cells compared with the negative control (**Figures 4C, D****)**. In addition, compared with normoxic conditions, hypoxia significantly downregulated the level of miR-455-5p and upregulated the expression of HOXA-AS3 (**Figures 4E, F****)**. We also found HOXA-AS3 levels were significantly downregulated in BC cells transfected with a miR-455-5p mimic but and upregulated by the miR-455-5p inhibitor (**Figure 4G****)**. Moreover, the expression of Notch1 was downregulated by the miR-455-5p mimic, while it was upregulated by the inhibitor **(****Figure 4H****)**. Luciferase reporter assay also confirmed the interaction between miR‐455-5p and Notch1 **(****Figures 4I, J****)**. The binding of miR-455-5p to HOXA-AS3 was shown to lead to a decrease in HOXA-AS3 transcript levels and a concomitant increase in the translation of the HOXA-AS3-associated mRNA Notch1. The data indicate that HOXA-AS3 may function as a competing endogenous RNA (ceRNA) of miR-455-5p to regulate Notch1 in BC, which in turn, may affect cisplatin sensitivity.

### miR-455-5p Mediates the Regulatory Effect of HOXA-AS3 on Cisplatin Sensitivity

**Figure 5:**
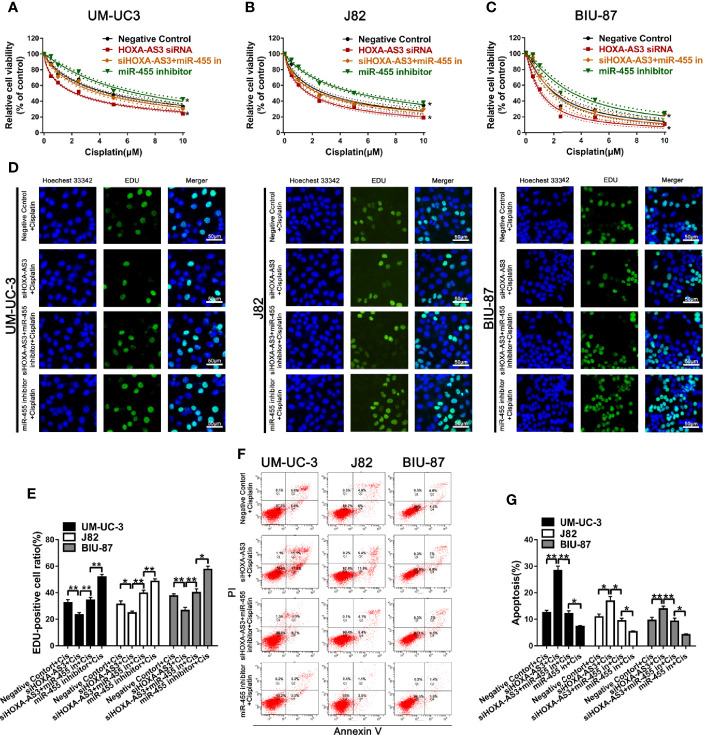
miR-455-5p mediates the regulatory effect of HOXA-AS3 on cisplatin sensitivity. **(A**–**C)** Viability of BC cells (CCK-8 assay) treated with different concentrations of cisplatin for 48 h (0, 0.625, 1.25, 2.5, 5, 10 μM) following transfection with HOXA-AS3 small interfering RNA (siRNA), miR-455-5p inhibitor, or HOXA-AX3 plus miR-455-5p inhibitor. *P < 0.05 *vs.* negative control. **(D**–**E)** Cell proliferation of BC cells (EdU staining) after treatment with cisplatin, or combined with HOXA-AS3 siRNA, or miR-455-5p inhibitor, or HOXA-AS3 siRNA plus miR-455-5p inhibitor. *P < 0.05, **P < 0.01, ***P < 0.001. **(F**, **G)** Apoptotic rate of BC cells in different treatment groups (Negative siRNA plus cisplatin, HOXA-AS3 siRNA plus cisplatin, HOXA-AS3 siRNA plus miR-455-5p plus cisplatin) detected by flow cytometry. *P < 0.05, **P < 0.01, ***P < 0.001.

Next, we explored whether miR-455-5p is involved in HOXA-AS3-mediated reduction in cisplatin sensitivity in BC cells. Using a CCK-8 (cell viability) assay, we showed miR-455-5p inhibition reduced the sensitivity of BC cells to the effects of cisplatin (**Figures 5A–C**). Meanwhile, HOXA-AS3 siRNA enhanced the sensitivity of BC cells to cisplatin, but these positive effects disappeared following the addition of the miR-455-5p inhibitor (**Figures 5A–C**). These results were consistent with those of EdU analysis **(****Figures 5D, E****)**. We also found that the miR-455-5p inhibitor reduced the apoptosis rate of BC cells after transfection with HOXA-AS3 siRNA along with cisplatin treatment **(****Figures 5F, G****)**. Therefore, both miR-455-5p and HOXA-AS3 are involved in modulating the cisplatin sensitivity of BC cells.

**Figure 6:**
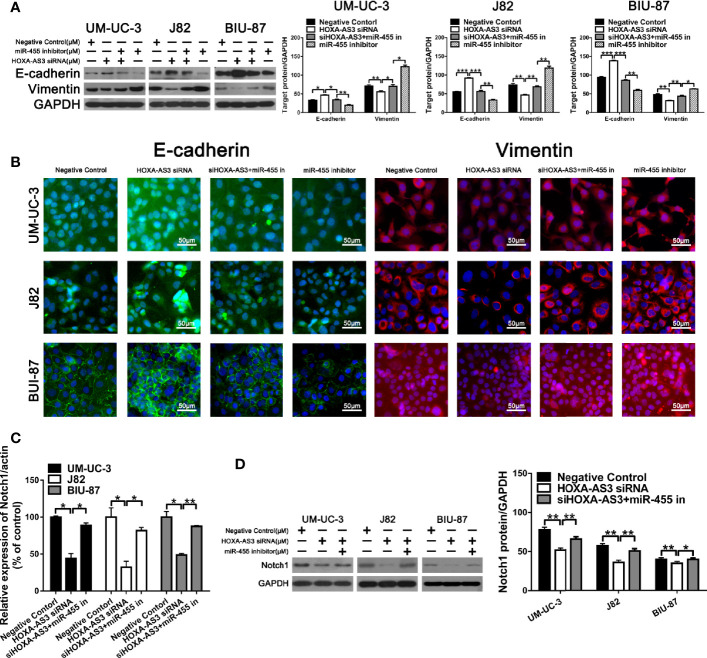
HOXA-AS3 small interfering RNA (siRNA) reversed miR-455-5p-induced EMT. **(A)** Western blot analysis of the expression of EMT-related proteins. *P < 0.05, **P < 0.01, ***P < 0.001. **(B)** Immunofluorescence analysis of E-cadherin and vimentin expression in bladder cancer cells transfected with a negative control, HOXA-AS3 siRNA, miR-455-5p inhibitor, or HOXA-AS3 siRNA combined with miR-455-5p inhibitor, followed by cisplatin treatment. **(C)** Quantitative RT-PCR analysis of Notch1 expression following treatment with HOXA-AS3 siRNA alone, HOXA-AS3 siRNA plus miR-455-5p inhibitor, or the negative control. *P < 0.05, **P < 0.01. **(D)** Western blot analysis of the expression of Notch1 following treatment with HOXA-AS3 siRNA alone, HOXA-AS3 siRNA plus miR-455-5p inhibitor, or the negative control. *P < 0.05, **P < 0.01.

In terms of its effects on EMT, we found HOXA-AS3 siRNA reversed the miR-455-5p-induced decrease of E-cadherin expression and increase of Vimentin expression **(****Figure 6A****)**. Immunofluorescence analysis of E-cadherin and vimentin expression showed consistent results **(****Figure 6B****)**. We also showed HOXA-AS3 siRNA significantly downregulated Notch1 expression (**Figure 6C**). Meanwhile, miR-455-5p restored Notch1 expression after HOXA-AS3 siRNA treatment **(****Figure 6D****)**. Together these findings reveal miR-455-5p mediates the regulatory effect of HOXA-AS3 on cisplatin sensitivity in BC cells.

### Suppression of HOXA-AS3 Enhances Cisplatin Sensitivity of BC Cells *In Vivo*

**Figure 7:**
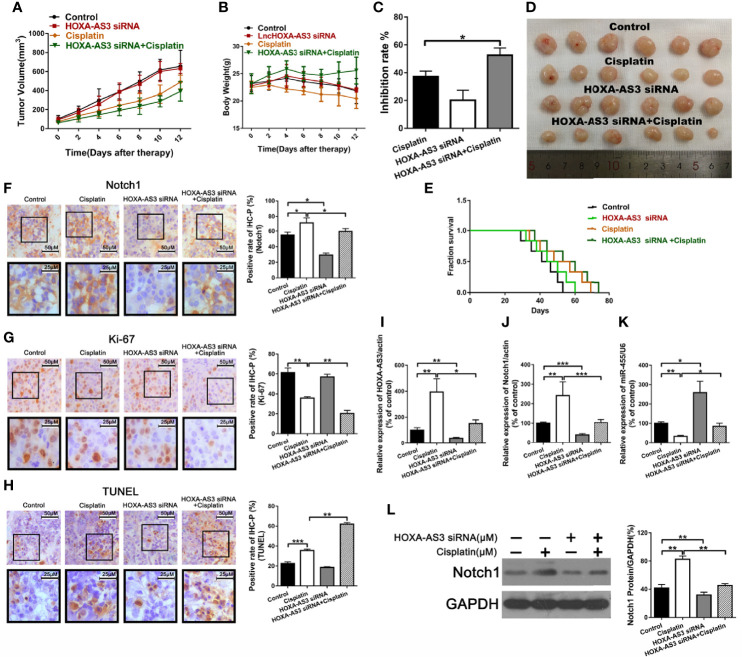
Suppression of HOXA-AS3 enhances the cisplatin sensitivity of bladder cancer cells *in vivo*. **(A)** Volume of tumor xenografts. **(B)** Body weights of mice measured on day 0–12. **(C)** Tumor inhibition rate. **(D)** Tumors isolated from nude mice. **(E)** Kaplan–Meier curves of mice in the saline groups. **(F–H)** Notch1 and Ki-67 staining in the different treatment groups (left) and rate of positive staining (right). *P < 0.05, **P < 0.01. **(G)** TUNEL assay of the level of apoptosis in the different treatment groups. **P < 0.01, ***P < 0.001. **(I–L)** HOXA-AS3, Notch1, and miR-455-5p expression levels in tumor tissues using qRT-PCR. *P < 0.05, **P < 0.01, ***P < 0.001. **(K)** Western blot analysis of HOXA-AS3 and Notch1 expression in tumor tissues. **P < 0.01.

To explore the effect of HOXA-AS3 on the sensitivity to cisplatin *in vivo*, we used UMUC-3 cells to establish a tumor xenograft mouse model. Mice were treated with normal saline, cisplatin, HOXA-AS3 siRNA, or HOXA-AS3 siRNA plus cisplatin. As shown in **Figures 7A, D**, the tumor volume was significantly reduced in mice treated with HOXA-AS3 siRNA plus cisplatin. In addition, the tumor inhibitory rate was higher in mice treated with HOXA-AS3 siRNA plus cisplatin than the other treatment groups **(****Figure 7C****)**. HOXA-AS3 siRNA could prolong the survival of tumor-bearing mice (**Figure 7E**). The body weight of mice on day 12 after tumor cell injection was significantly lower in the cisplatin-treated group than in the other three groups; however, the body weight was higher in mice treated with HOXA-AS3 siRNA plus cisplatin than in those treated with cisplatin alone **(****Figure 7B****)**.

We also showed that treatment with HOXA-AS3 siRNA plus cisplatin downregulated the cisplatin-induced elevation of Notch1 expression **(****Figure 7F****)**. In addition, treatment with HOXA-AS3 siRNA plus cisplatin significantly decreased tumor cell proliferation and increased tumor cell apoptosis compared to the other three groups **(****Figures 7G, H****)**. Furthermore, treatment with HOXA-AS3 siRNA plus cisplatin significantly downregulated the level of HOXA-AS3, Notch1, and miR-455-5p, as revealed by qRT-PCR **(****Figures 7I–L****)**. Notch1 protein expression was also reduced following treatment with HOXA-AS3 siRNA plus cisplatin, compared with the cisplatin group, as revealed by western blot analysis **(****Figure 7K****)**. These data support our findings from the *in vitro* studies and, thus, confirm the *in vivo* function of HOXA-AS3.

## Discussion

HOXA-AS3 was previously shown to function as an oncogene and was upregulated in various cancers including glioma tissues, NSCLC, and HCC (21, 31, 34). Similarly, we found HOXA-AS3 was upregulated in BC tumor tissues and BC cell lines. The high HOXA-AS3 expression in 30 bladder cancer patients was closely related to large tumor size, the advanced TNM stage and invasion(muscle). Moreover, the expression of HOXA-AS3 in BC cells was correlated with the sensitivity to cisplatin, indicating that HOXA-AS3 might be a useful biomarker to predict sensitivity to cisplatin in BC. Our *in vitro* and *in vivo* experiments confirmed HOXA-AS3 inhibition could reduce BC cell viability and tumor growth. We also clarified the role of HOXA-AS3 in mediating cisplatin resistance in BC cells. Overall, our findings indicate that targeting HOXA-AS3 could be useful for optimizing cisplatin treatment in BC.

Extensive evidence has revealed that lncRNAs could serve as ceRNAs or molecular sponges to directly interact with and negatively regulate miRNAs, thus modulating the expression of specific genes targeted by miRNAs (11, 37). For instance, overexpression of the lncRNA UBE2R2-AS1 promoted glioma cell apoptosis *via* targeting the miR-877-3p/TLR4 axis (38). In addition, suppression of the lncRNA MALAT1 enhanced cisplatin sensitivity *via* regulating the miR-101-3p/VEGF-C pathway in BC (20). Similarly, we showed miR-455-5p was a direct target of HOXA-AS3: HOXA-AS3 siRNA transfection increased miR-455-5p levels, while HOXA-AS3 transfection decreased them. Indeed, a growing number of studies have demonstrated miRNAs participate in regulating drug sensitivity in several cancers, including BC. Therefore, we explored the effects of miR-455-5p on cisplatin resistance in BC.

Previous studies have shown miR-455-5p can function as either a tumor promoter or suppressor in various cancers. For example, miR-455-5p was downregulated in colorectal carcinoma and gastric cancer, but was upregulated in NSCLC (39–41). We confirmed that miR-455-5p inhibition could decrease the sensitivity of BC cells to cisplatin, as well as promote BC cell proliferation and reduce apoptosis, indicating miR-455-5p acts as a tumor suppressor in BC. We also found that combining an miR-455-5p inhibitor with HOXA-AS3 siRNA alleviated the effect of HOXA-AS3 siRNA on cisplatin sensitivity. These findings indicate HOXA-AS3 regulates cisplatin sensitivity *via* regulation of miR-455-5p in BC.

Not only is EMT is strongly correlated with drug resistance, the emergence of drug resistance may also occur as a result of EMT (42, 43). Emerging evidence indicates that Notch signaling plays an important role in cell proliferation and apoptosis, which are involved in the development and functioning of many organs (44). Activation of the Notch1 pathway has been commonly observed in many human malignancies including PC (45). Downregulation of the lncRNA XIST in NSCLC cells could suppress cell proliferation and TGF-β1-induced EMT through activation of the Notch1 pathway *via* regulation of miR-137 (46). In addition, recent studies show HOXA-AS3 may regulate cisplatin resistance and metastasis in NSCLC and HCC cells *via* modulating EMT (31, 34). Similarly, we showed that HOXA-AS3 siRNA enhanced cisplatin sensitivity in BC *via* modulating EMT, and confirmed that Notch1 was mediating these effects.

In conclusion, HOAX-AS3 inhibition may suppress tumor progression and enhance sensitivity to cisplatin in BC *via* modulation of the miR-455-5p-Notch1 axis. As the HOXA-AS3–miR-455-5p–Notch1 network plays a central role in cisplatin resistance, targeting HOXA-AS3 may help optimize the efficacy of cisplatin treatment for BC in the future.

## Data Availability Statement

The original contributions presented in the study are included in the article/**Supplementary Material**, further inquiries can be directed to the corresponding authors.

## Ethics Statement

All experimental protocols were approved by the Medical Ethics Committee of the First Affiliated Hospital of Zhejiang University. The experimental procedures conformed to the National Institutes of Health Guide for Care and Use of Laboratory Animals (NIH Publications, No. 8023, revised 1978).

## Author Contributions

WC, DC, and JC conceived the idea. DC and SX performed the experiments. YCu and YCa collected the data. LL analyzed the data. HY created the figures. DC conducted the literature search. WC and DC wrote the manuscript. YW revised the manuscript. All authors contributed to the article and approved the submitted version.

## Funding

This study was funded by the Natural Science Foundation of China (grant no. 81802085) and the Natural Science Foundation of Zhejiang Province (grant no. LY21H160033, LR20H160001), Key R&D projects of Zhejiang Province (2020C03G5263593), Zhejiang Provincial Ten Thousand Plan for Young Top Talents (2018), Training objects of health innovative talents of Zhejiang Health (2018), Key Project Co-constructed by Zhejiang Province and Ministry (WKJ-ZJ-1916), Zhejiang Provincial Traditional Chinese Medicine Science and Technology Project (2020ZZ004).

## Conflict of Interest

The authors declare that the research was conducted in the absence of any commercial or financial relationships that could be construed as a potential conflict of interest.

## Supplementary Material

Supplementary Figure 1 **(A, B)**. HOXA-AS3 expression in bladder cancer cells quantified by qRT-PCR after treatment with different concentrations of cisplatin **(A)** or for a different length of time **(B)**.

Supplementary Figure 2 **(A–D)**. CC-K assay analysis cell viability transfection with or without HOSA-AS3 plasmid and combined with cisplatin in BC cells, the expression of HOXA-AS3 was determined by qRT-PCR. *P<0.05 vs Control.

Supplementary Figure 3 A.QRT-PCR determined the expression of miRNA following treatment with or without cisplatin in BC cells.
