# Supplementary figures and images for: Reduction of Bladder Cancer Chemosensitivity Induced by the Effect of HOXA-AS3 as a ceRNA for miR-455-5p That Upregulates Notch1
Source: Front Oncol. 2021 Feb 12;10:572672. doi: 10.3389/fonc.2020.572672 (PMC7907523; doi:10.3389/fonc.2020.572672)

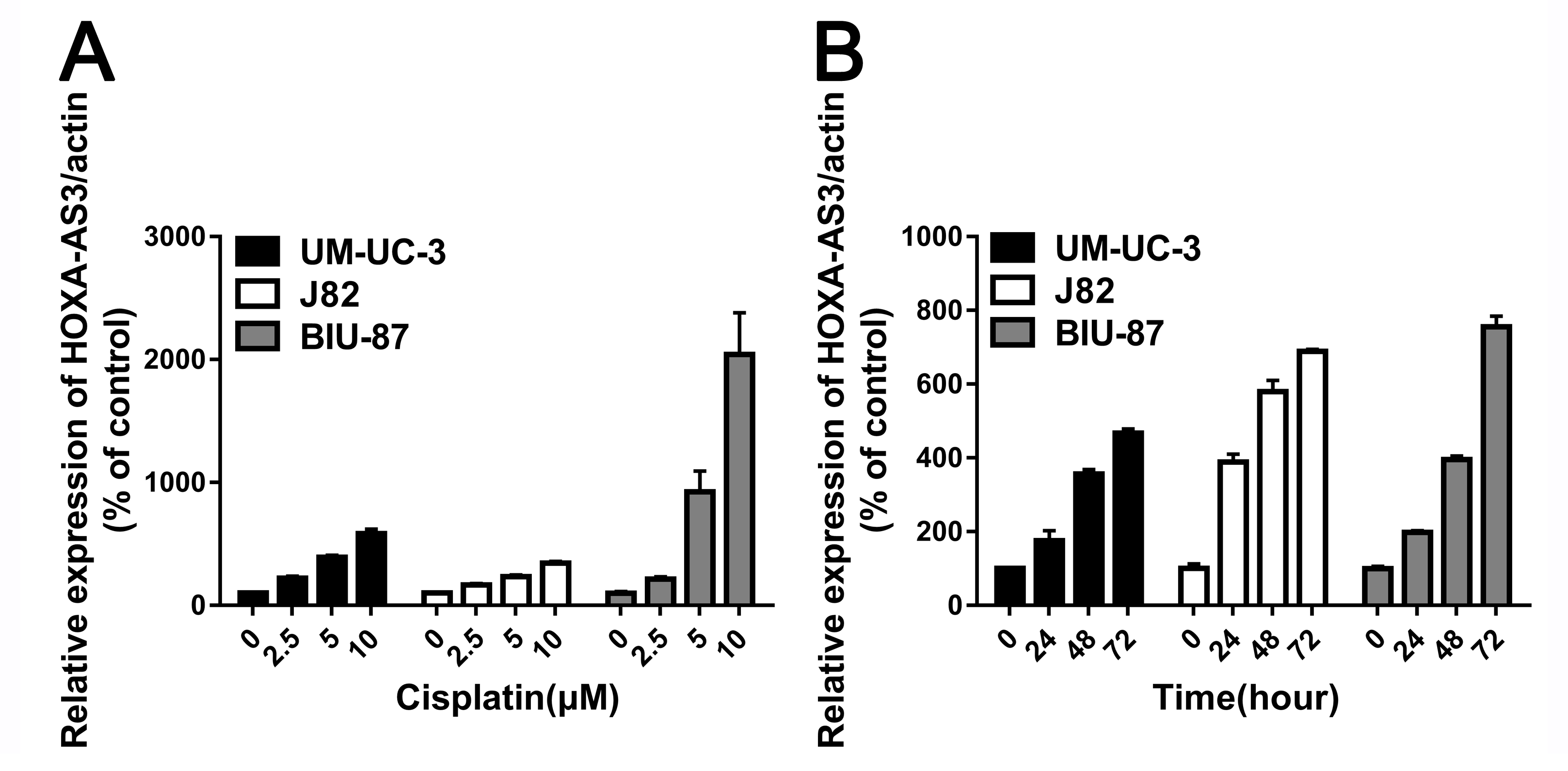

Supplement: Supplementary Figure 1 — (A, B). HOXA-AS3 expression in bladder cancer cells quantified by qRT-PCR after treatment with different concentrations of cisplatin (A) or for a different length of time (B). [file Image_1.tif]

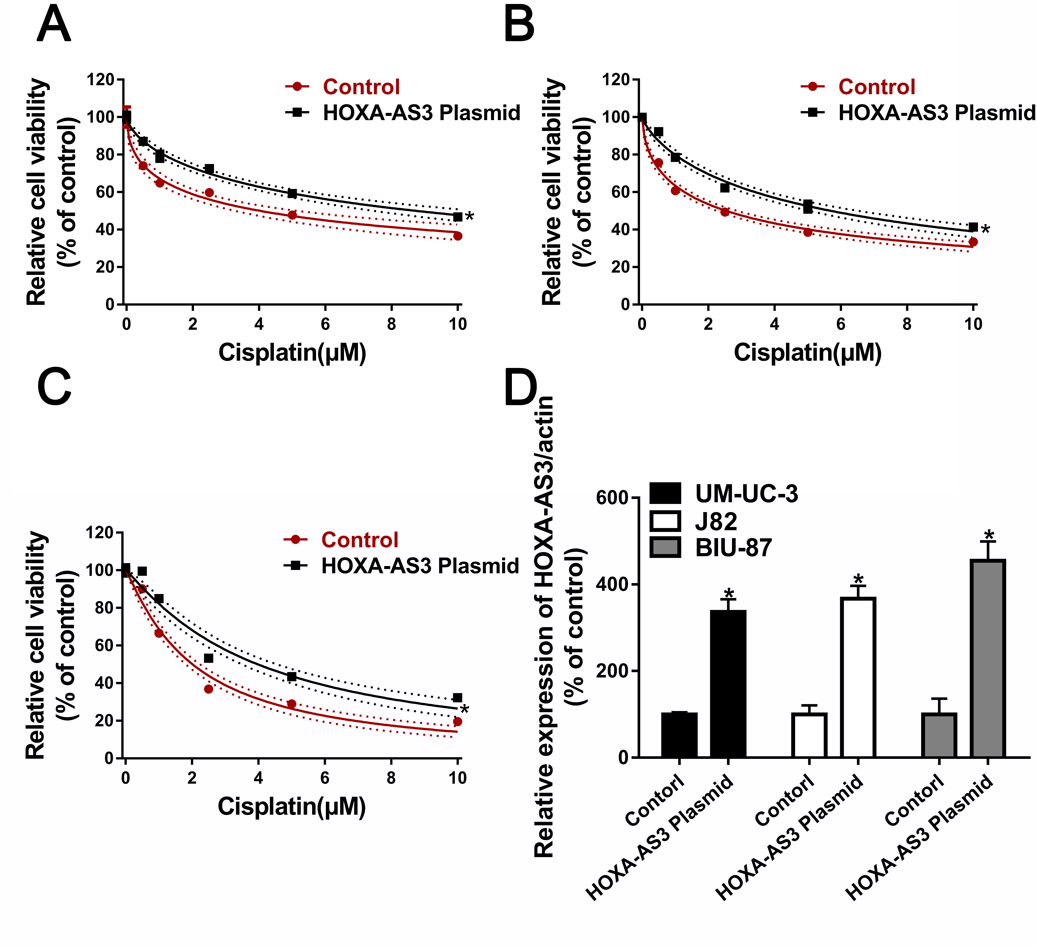

Supplement: Supplementary Figure 2 — (A–D). CC-K assay analysis cell viability transfection with or without HOSA-AS3 plasmid and combined with cisplatin in BC cells, the expression of HOXA-AS3 was determined by qRT-PCR. *P<0.05 vs Control. [file Image_2.tif]

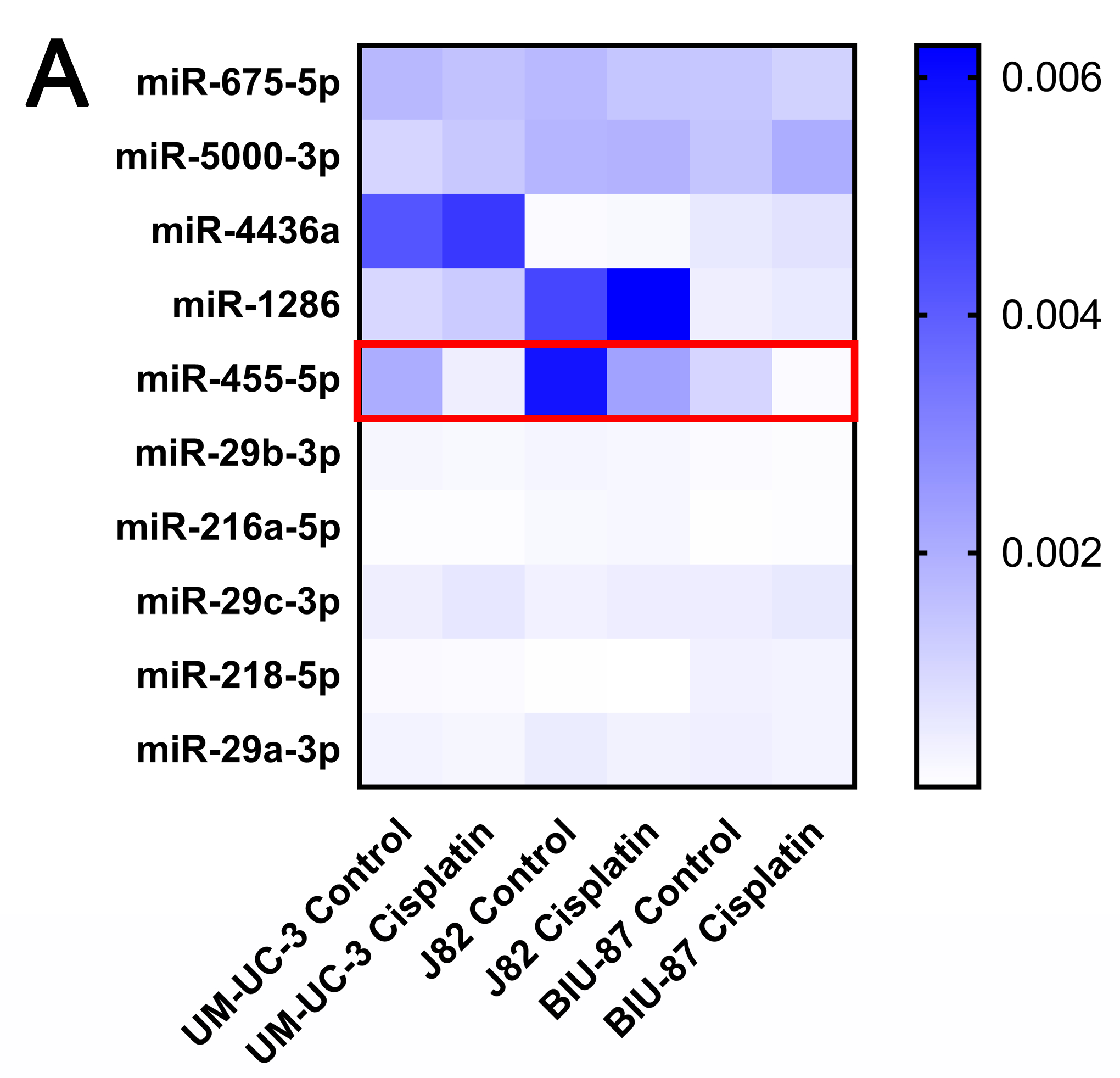

Supplement: Supplementary Figure 3 — A.QRT-PCR determined the expression of miRNA following treatment with or without cisplatin in BC cells. [file Image_3.tif]
